# Ubiquitin Binding Protein 2-Like (UBAP2L): is it so NICE After All?

**DOI:** 10.3389/fcell.2022.931115

**Published:** 2022-06-20

**Authors:** Lucile Guerber, Evanthia Pangou, Izabela Sumara

**Affiliations:** ^1^ Institut de Génétique et de Biologie Moléculaire et Cellulaire (IGBMC), Illkirch, France; ^2^ Centre National de la Recherche Scientifique UMR 7104, Strasbourg, France; ^3^ Institut National de la Santé et de la Recherche Médicale U964, Strasbourg, France; ^4^ Université de Strasbourg, Strasbourg, France

**Keywords:** UBAP2L, mitosis, cancer, ubiquitin, stress signaling

## Abstract

Ubiquitin Binding Protein 2-like (UBAP2L, also known as NICE-4) is a ubiquitin- and RNA-binding protein, highly conserved in metazoans. Despite its abundance, its functions have only recently started to be characterized. Several studies have demonstrated the crucial involvement of UBAP2L in various cellular processes such as cell cycle regulation, stem cell activity and stress-response signaling. In addition, UBAP2L has recently emerged as a master regulator of growth and proliferation in several human cancers, where it is suggested to display oncogenic properties. Given that this versatile protein is involved in the regulation of multiple and distinct cellular pathways, actively contributing to the maintenance of cell homeostasis and survival, UBAP2L might represent a good candidate for future therapeutic studies. In this review, we discuss the current knowledge and latest advances on elucidating UBAP2L cellular functions, with an aim to highlight the importance of targeting UBAP2L for future therapies.

## Introduction

Ubiquitin Associated Protein 2-Like (UBAP2L) or NICE-4 is a highly conserved protein in vertebrates (Chang et al., 2018). Encoded by the KIAA0144 gene located on the chromosomal region 1q21, NICE-4 was originally identified by Marenholz and colleagues in an effort to discover new Human Epidermal Differentiation Complex (EDC)-encoded genes (Marenholz et al., 2001). Five different isoforms produced by alternative splicing have been reported for UBAP2L, that are broadly expressed in nearly all tissues. Despite its abundant expression, UBAP2L has only recently attracted attention of broad scientific community which led to the discovery of its highly versatile roles. Interestingly, UBAP2L orthologs have been identified in metazoans such as Prion-like (Q/N-rich)-domain-bearing protein (PQN-59) in *Caenorhabditis elegans* and lingerer in *Drosophila melanogaster* (Uhlén et al., 2015).

**FIGURE 1:**
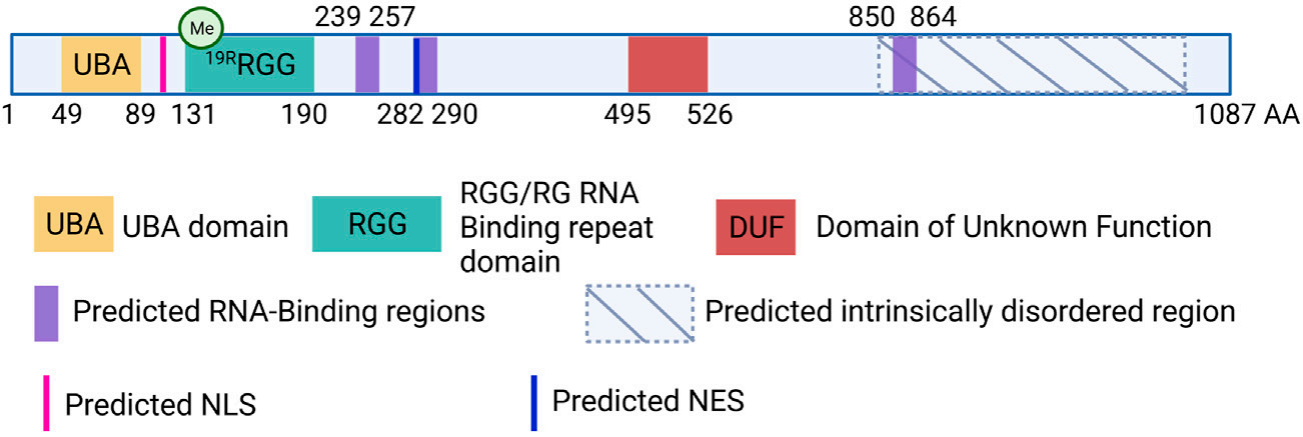
Schematic view of the human UBAP2L protein and its domain organization. UBAP2L (1087 AA) is composed of a Ubiquitin-associated domain (UBA, yellow), an Arginine-Glycine-Glycine (RGG, green) domain and a Domain of Unknow Function (DUF, red). Additional RNA-binding regions have been predicted and are painted in purple. Moreover, UBAP2L is predicted to contain several Nuclear Localization Signals (NLS) and Nuclear Export Signals (NES) (pink and dark blue respectively). Several UBAP2L regions have been proposed to be intrinsically disordered (IDR), and prone to liquid-liquid phase-separation. The most conserved ones are shown with hatched lines. It is important to note that for simplicity we chose to depict only some of the predicted NLS, NES and IDR regions of UBAP2L in the scheme, and that this does not exclude the existence of other similar motifs or regions. Similarly, documented methylation modification on 19 different arginines (19R) present within the RGG domain has been indicated schematically.

UBAP2L is a 1,087 amino-acid (aa)-long protein, structurally composed of a N-terminal Ubiquitin-Associated Domain (UBA; aa 49-89), an Arginine-Glycine-Glycine (RGG; aa 131-190) domain and three predicted RNA-Binding regions (aa 239-257, aa 282-290 and aa 850-864) (Castello et al., 2016) (Figure 1). SILAC analysis demonstrated that UBAP2L cofractionates with ubiquitin in aggregates following proteasomal inhibition, emphasizing the functionality of its UBA domain (Wilde et al., 2011). Moreover, ribosome profiling studies demonstrated that UBAP2L promotes translation of target mRNAs suggesting that it can act as a ribosome-binding protein essential for protein synthesis (Luo et al., 2020). In addition, UBAP2L harbors a Domain of Unknown Function (DUF; aa 495-526). Prediction tools have unraveled several disordered regions prone to undergo Liquid-Liquid Phase Separation (LLPS) as well as several Nuclear Localization Signals (NLS) and Nuclear Export Signals (NES), suggesting that UBAP2L is shuttling between the cytoplasm and the nucleus. Such atypical domain organization classifies UBAP2L in both Ubiquitin-binding and RNA-binding proteins superfamilies, highlighting its potential involvement in a plethora of cellular processes.

Although UBAP2L was initially described as an interactor of the Human Zona Pellucida Sperm-binding protein 3 (ZP3) (Naz and Dhandapani, 2010), during the last decade additional studies have demonstrated its direct involvement in cell growth, mitotic progression, stem cell activity, apoptosis and stress response signaling (Bordeleau et al., 2014; Li and Huang, 2014; Chai et al., 2016; Maeda et al., 2016; Youn et al., 2018; Huang et al., 2020). Moreover, UBAP2L is overexpressed in different types of cancer, displaying oncogenic potential and often correlating with poor prognosis (Li and Huang, 2014; Zhao et al., 2015; Bai et al., 2016; Chai et al., 2016; Aucagne et al., 2017; He et al., 2018; Yoshida et al., 2020; Guan et al., 2021). Of note, UBAP2L KO mice die before birth or within minutes after surgical delivery from acute respiratory failure, demonstrating that UBAP2L holds housekeeping functions, essential for living organisms (Aucagne et al., 2017). This review discusses the current knowledge and the latest advances on elucidating NICE-4 cellular functions, with an aim to highlight the importance of targeting NICE4 for future therapies.

## UBAP2L and Cellular Homeostasis

### UBAP2L and Stem Cell Activity

As mentioned above, UBAP2L KO mice die prematurely, pointing to a potential role for UBAP2L during development. Interestingly, in *C. elegans*, PQN-59 has been shown to modulate gene expression thus playing a key role in cell fate specification during development (Carlston et al., 2021). In an embryo, undifferentiated cells, called stem cells, give rise to one or several types of differentiated cells which later form mature tissues and organs. UBAP2L was proposed to be modified by O-Linked N-Acetylglucosamine (O-Glc-NAc) in mouse MC3T3E1 differentiating osteoblasts (Nagel et al., 2013). Interestingly, UBAP2L is found enriched in osteoblasts and as such it is used as an osteoblast marker (Guan et al., 2021). More globally, UBAP2L expression is increased in other types of undifferentiated cells such as mouse and human hematopoietic and leukemic stem cells. In the above study, Bordeleau and colleagues propose a model in which UBAP2L forms a complex with the Polycomb group (PcG) proteins BMI1 and Ring Finger Protein 2 (RNF2), thereby regulating long-term repopulating hematopoietic stem cells (LT-HSCs) independently of Ink4a/Arf locus repression, a popular target of BMI1. The authors suggest that at least two Polycomb-repressive complexes can assemble in order to regulate HSC function, which are distinguishable by the presence or the absence of UBAP2L (Bordeleau et al., 2014). Further investigations are needed in order to elucidate UBAP2L’s precise role as part of the Polycomb complex since the exact mechanism has not been fully understood yet. A partial answer has been provided by Lin et al. who used rat bone marrow mesenchymal stem cells (BMSCs) overexpressing UBAP2L to transplant it to rats suffering from semi-sectioned spinal cord injury (SPI) and to monitor the recovery of the injured tissue (Lin et al., 2018). UBAP2L overexpressing cells exhibited stronger neuronal differentiation potential, which led to faster spinal cord function recovery. Mechanistically, UBAP2L overexpression results in increased expression of the cell cycle related protein cyclin D1 and of p38 MAPK, and more importantly to decreased expression of Caspase 3, a key apoptotic factor responsible for the majority of post-SCI neuronal death (Yu and Fehlings, 2011). Overall, the authors propose that UBAP2L overexpression in BMSCs promotes neuronal proliferation and survival, limits contingent damage like post-SCI inflammation and eventually leads to SCI repair (Lin et al., 2018). Given that the UBAP2L locus has been associated with other neuronal disorders such as bipolar or anorexia nervosa disorders (eQTLGen Consortium et al., 2019; Iranzo-Tatay et al., 2022), it would be of great interest to further investigate its potential role in the development of other neurological and aging-related neurodegenerative diseases.

### UBAP2L and Cell Division

In eukaryotes, mitosis is a crucial process which needs to be tightly regulated in time and space to allow for faithful division of a mother cell into two identical daughter cells (McIntosh, 2016). UBAP2L has been proposed to regulate cell division. Its depletion impairs chromosome alignment during metaphase and potentiates Spindle Assembly Checkpoint (SAC) response. Chromosome misalignment phenotypes upon UBAP2L depletion occur due to the disruption of stable k-fibers, suggesting defects in proper microtubule-kinetochore (MT-KT) attachment, which in turn hinders proper chromosome segregation and mitosis completion (Maeda et al., 2016). Maeda and colleagues further showed that UBAP2L RGG/RG domain is responsible for the multi- and micronucleation phenotypes observed in UBAP2L downregulated HeLa cells and more importantly that this function is mediated by the methylation of the arginines within the RGG/RG domain by the methyl-transferase PRMT1. Although the construct lacking this post-translational modification is properly localized at the spindle, it cannot rescue chromosome misalignment during metaphase observed in UBAP2L depleted cells suggesting that UBAP2L RGG/RG domain methylation is essential for proper MT-KT attachments, accurate chromosome distribution and proper mitotic progression. Consistently, UBAP2L depletion leads to an enrichment of G2/Mitotic (G2/M) population in HeLa cells (Maeda et al., 2016), in ZR-75-30 and in T-47D breast cancer cells (He et al., 2018) and in DU145 prostate cancer cells (Li and Huang, 2014) pointing to an important role of UBAP2L as a cell cycle regulator.

### UBAP2L and Stress Signaling

An interesting feature of UBAP2L protein is its ability to aggregate and to regulate protein synthesis as indicated above (Wilde et al., 2011; Luo et al., 2020). mRNA turnover and protection under stress conditions have been associated with the formation of Stress Granules (SG) (Parker and Sheth, 2007). In an attempt to identify new components and/or regulators of cytosolic RNA granules, Youn and colleagues performed proximity-based proteomics and identified UBAP2L as a critical factor for efficient SG assembly following stress induced by the arsenite treatment. Importantly, the DUF domain of UBAP2L containing an phenylalanine-glycine phenylalanine-glycine (FG-FG) motif is critical for G3BP1 (Ras GTPase-activating protein-binding protein 1) recognition and binding in flies (Baumgartner et al., 2013) and is responsible for G3BP1 assembly in HeLa cells. In contrast, UBA and RGG domains of UBAP2L seem to be dispensable for SG formation (Youn et al., 2018). Subsequent studies by another group demonstrated the crucial role of the RGG domain of UBAP2L for SG competence under stress-null and stress conditions (Huang et al., 2020). More precisely, under stress conditions, UBAP2L methylation by PRMT1 is decreased, enabling UBAP2L’s interaction with SG components and subsequently promoting SG assembly. The authors show that UBAP2L’s DUF domain is still very important for G3BP1/2 NTF2-like domain binding and localization. In fact, depletion of the DUF domain promotes UBAP2L shuttling from the cytoplasm to the nucleus, impeding its interaction with G3BP1/2 and consecutively abolishes SG formation (Huang et al., 2020). Further work from Gotta group, propose that UBAP2L forms SG cores to which G3BP1 is subsequently recruited to allow for SG maturation, suggesting that UBAP2L acts upstream of G3BP1 in SG nucleation (Cirillo et al., 2020). Intriguingly, this phenomenon seems to be specific to human cells as a recent study from the same group established that PQN-59 and GTBP-1 (the human UBAP2L and G3BP1/2 orthologs respectively) are not essential for SG assembly in C. elegans (Abbatemarco et al., 2021). Interestingly, additional types of subcellular complexes can be assembled under stress conditions. Among them, the nuclear “twins” of SG are called paraspeckles (PS). These ribonucleoproteins (RNP) granules assemble around the long noncoding RNA (lncRNA) NEAT1 (Fox et al., 2018). Upon stress induction, SGs regulate PSs assembly via the sequestration of important negative regulators of PS formation such as UBAP2L (An et al., 2019). For the moment, we still lack sufficient knowledge to explain the molecular mechanism behind this regulation and it would be important to understand if and how UBAP2L acts as a global regulator of stress-induced complex assemblies, in addition to its well-established role in SGs.

## UBAP2L and Cancer

**FIGURE 2:**
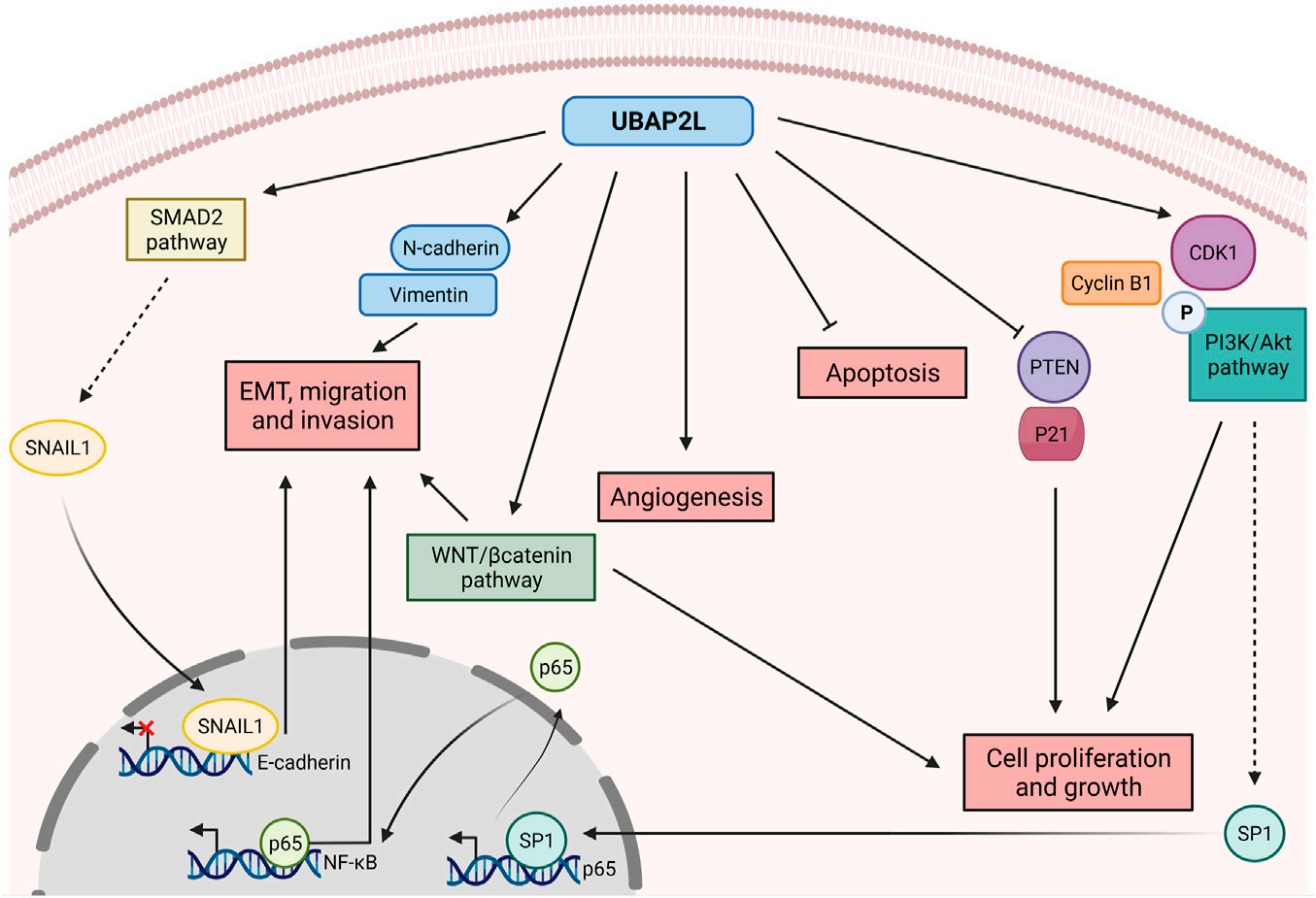
Versatile roles of UBAP2L in promoting cancer disease. UBAP2L upregulates key cell cycle regulators such as CyclinB1, CDK1 and the PI3K/Akt pathway, while it inhibits the expression of tumor suppressors such as PTEN and P21, thereby promoting cell proliferation and growth. PI3K/Akt activation enhances SP1 levels which in turn activates P65 expression, thereby activating NF-κB pathway and favoring epithelial-mesenchymal transition (EMT), migration and invasion. The metastatic potential of UBAP2L-overexpressing cells is also sustained by the activation of the SMAD2 pathway, triggering the transcriptional repressor SNAIL1 to the E-cadherin promoter, shutting down its expression. Cancer cells overexpressing UBAP2L are characterized by hyperactivation of the WNT/βcatenin pathway and by upregulation of mesenchymal factors such as N-cadherin and Vimentin, resulting in increased invasion and proliferation. Finally, UBAP2L favors tumor vascularization while inhibiting cancer cells apoptosis. Overall, UBAP2L promotes cancer progression by regulating various axes of tumorigenesis known as the hallmarks of cancer.

Recent work has demonstrated that UBAP2L is overexpressed in a variety of cancers and as such it has gained significant attention of researchers over the past years. Although its aberrant expression is a common feature of very different types of tumors, the way UBAP2L acts to promote carcinogenesis appears to be highly variable (Figure 2), highlighting UBAP2L’s versatile functions not only in healthy tissues but also under pathological conditions. As mentioned above, UBAP2L is broadly expressed in almost all tissues. Likewise, this abundance is also found and exacerbated in distinct tumor types such as prostate, breast, uterine, cervical, non-small cell lung and gastric cancers, glioma, colorectal and hepatocellular carcinoma (HCC) and lung adenocarcinoma (Li and Huang, 2014; Zhao et al., 2015; Bai et al., 2016; Chai et al., 2016; Aucagne et al., 2017; Wang et al., 2017; Ye et al., 2017; He et al., 2018; Li et al., 2018; Lin et al., 2018; Pan et al., 2020; Yoshida et al., 2020; Guan et al., 2021; Yang et al., 2021; Li et al., 2022). In nearly all cited cancer studies, UBAP2L is suggested to act as an oncogene promoting cancer cell proliferation and growth *in vitro* and *in vivo*, thus providing an explanation to the existing negative correlation between UBAP2L expression and patients’ prognosis.

### UBAP2L Promotes Cell Proliferation and Growth

In prostate, breast cancers and HCC, UBAP2L depletion leads to an accumulation of G2/M cell population (Li and Huang, 2014; He et al., 2018; Li et al., 2018), whereas it was shown to increase the G0/G1 cells rate in Glioma and colorectal carcinoma, suggesting that UBAP2L may act during several cell cycle stages (Zhao et al., 2015; Chai et al., 2016). Additionally, UBAP2L is responsible for the multifaceted regulation of tumors’ cellular and molecular properties in order to promote cellular survival as well as migration. Compelling evidence suggests that oncogenic pathways rely on the establishment of a suitable micro-environment that provides nutrients and supports tumor development and survival as elegantly summarized in 2011 (Hanahan and Weinberg, 2011). Intriguingly, UBAP2L seems to be involved in the regulation of several hallmarks of cancer.

Firstly, as mentioned above, UBAP2L sustains cell proliferation potentially via the regulation of cell cycle signaling pathways. For instance, it has been observed that knockdown of UBAP2L increases p21 and decreases CDK1 and CyclinB1 expression in breast cancer cells (He et al., 2018). This observation was further confirmed in HCC in a study showing a gene enrichment analysis after UBAP2L depletion. As previously demonstrated, the authors found PTEN and p21 among the most upregulated genes, while CDK1, CyclinB1, p-PI3K and p-AKT were among the most downregulated genes following UBAP2L silencing (Li et al., 2018). The signaling pathways downstream of PTEN, TP53 and PI3K/Akt are commonly dysregulated and hijacked in cancerous cells in order to promote their growth as extensively reviewed in the past years (Hollander et al., 2011; Khemlina et al., 2017; Levine, 2020). Of particular interest, the PI3K/Akt pathway is implicated in a broad range of cellular processes including cell proliferation but also apoptosis, angiogenesis, replicative immortality, invasion and metastasis, pointing out to UBAP2L oncogene as a golden target for future anti-cancer therapies (Lien et al., 2017). The molecular mechanism of how UBAP2L might regulate the PI3K/Akt pathway can be partially explained by a study suggesting that UBAP2L activates the PI3K/Akt pathway by promoting a phosphorylation cascade which in turn triggers SP1 binding to P65 promoter, inducing its expression. UBAP2L enables P65 translocation into the nucleus and possibly activates NF-KB (Li et al., 2022), a pathway strongly associated to cancer progression (Zinatizadeh et al., 2021). However, further efforts are required in order to dissect how UBAP2L precisely regulates signaling pathways to enable cancer progression.

### UBAP2L Promotes Epithelial-Mesenchymal Transition, Migration, Invasion and Metastasis

An additional common feature of cancer cells is the ability to undergo epithelial-mesenchymal transition (EMT) as a means to promote effective invasion and metastasis (Hanahan and Weinberg, 2011). Interestingly, wound-healing assays of HCC cells lacking UBAP2L, revealed defects in migration and invasion. Consistently, cells lacking UBAP2L harbor increased epithelial (E-cadherin, CK-18) and decreased mesenchymal markers (N-cadherin, vimentin) (Ye et al., 2017), highlighting UBAP2L’s crucial role in regulating the metastatic potential of cancer cells. In addition to HCC, the promotion of EMT by UBAP2L has also been reported in prostate, lung and gastric cancers (Li and Huang, 2014; Aucagne et al., 2017; Lin et al., 2021). Complementary studies verified these conclusions *in vivo* where inhibition of UBAP2L led to defective cancer invasion in xenografts (Guan et al., 2021). In addition, mice injected with Ubap2l−/− A549 cells show less nodules in their lungs, lighter lungs and increased survival 3 weeks after injection in contrast to mice injected with Ubap2l+/+ A549 cells (Aucagne et al., 2017), while the opposite result is observed in gastric cancer when UBAP2L is overexpressed (Li et al., 2022). Finally, it was recently suggested that UBAP2L positively regulates the expression of the transcriptional repressor SNAIL1 *via* the SMAD2 signaling pathway which subsequently binds to and inhibits the promoter of E-cadherin, hindering the expression of this epithelial marker in favor of mesenchymal ones, ultimately leading to EMT, invasion and metastasis (Ye et al., 2017).

As previously discussed, cancer cells must use many diverse strategies to escape the cellular surveillance mechanisms in order to survive and migrate. To this end, most of the signaling pathways exploited by normal cells have to be hijacked, to favor cancer progression. For example, components of the Wnt/β-catenin signaling which is a highly conserved pathway regulating fundamental developmental processes, has been frequently observed to be mutated in cancer (Nusse and Clevers, 2017). Not surprisingly, UBAP2L has been proposed to activate the Wnt/β-catenin signaling cascade in gastric cancer cells, leading to the expression of downstream pathway targets, known to be implicated in tumorigenesis and metastasis (Yook et al., 2006; Liu et al., 2010; Damsky et al., 2011; Lin et al., 2021). However, the precise molecular mechanisms driving UBAP2L’s oncogenic potential are not yet defined. UBAP2L has been reported as a BMI1 interactor as cited before (Bordeleau et al., 2014). Although BMI1 is essential for the activity of hematopoietic stem cells, it has also been suggested as a Wnt signaling activator by regulating the Wnt antagonist IDAX (Yu et al., 2018). Therefore, one hypothesis that could be further explored, might be that Wnt/β-catenin hyperactivity in UBAP2L-overexpressing tumors could be attributed to UBAP2L/BMI1 interaction.

### UBAP2L Prevents Apoptosis of Cancer Cells and Promotes Tumor Vascularization

Cancer cells must acquire resistance to cellular death to ensure their survival and expansion (Hanahan and Weinberg, 2011). In this context, UBAP2L is suggested to act as an anti-apoptotic factor possibly by regulating, through yet unknown mechanisms, the expression of crucial apoptotic factors such as Bad/Bax and the cleavage of PARP and caspase 3 (Li and Huang, 2014; Chai et al., 2016). Bypassing all checkpoints employed by the cellular machinery is a challenge for cancer cells. Nevertheless, tumor microenvironment is crucial for proper cancer dissemination across tissues. For instance, cancerous cells require a certain amount of nutrients and oxygen to function properly and these components are efficiently brought to the cells only if the tumor is properly vascularized. Interestingly, samples from HCC patients revealed a positive correlation between UBAP2L and VEGF expression, a crucial protein for angiogenesis. Consistently, micro vessel density was also found to be increased in UBAP2L overexpressing tumors (Wang et al., 2017) and a complementary study from another laboratory reported that UBAP2L downregulation decreases the average vascular length and number of vascular branches (Li et al., 2018), once more pointing to a potential role for UBAP2L in favoring angiogenesis.

## UBAP2L and RNAs

Incremental studies were conducted on microRNAs (miRNAs), small nucleotides duplexes which post-transcriptionally regulate gene expression of their targets, being involved in general biological processes such as cell proliferation, apoptosis or brain development among others (Ambros, 2004). Intriguingly, UBAP2L was demonstrated to be targeted by different miRNA. First, in non-small cell lung cancer (NSCLC), miR-19a-3p directly inhibits UBAP2L, resulting in similar phenotypes as those observed upon UBAP2L downregulation, mainly inhibition of cell proliferation, migration and invasion (Pan et al., 2020). Similarly, UBAP2L was silenced by miR-148b-3p in gastric cancer cells leading to the same phenotypes as in NSCLC (Lin et al., 2021). Interestingly, the UBAP2L ortholog PQN-59 stabilizes several miRNAs involved in various cellular functions and interacts with RNA metabolism, transcription and translation cellular components similarly to UBAP2L, highlighting the importance of this protein in RNA regulation (Carlston et al., 2021). Supporting this hypothesis, UBAP2L localizes to stress granules and P-bodies under certain conditions, two structures highly linked to RNA turnover, miRNA or gene expression regulation (Leung et al., 2006).

### Concluding Remarks

Conclusively, although UBAP2L has been identified more than 20 years ago, its extremely versatile roles in various signaling pathways have been elucidated only recently. It would therefore be fascinating that future studies address the underlying precise molecular mechanisms that govern and direct UBAP2L’s functions towards such distinct signaling nodes to ensure cellular homeostasis. Our review aimed at highlighting the growing evidence on the oncogenic potential of UBAP2L that may identify UBAP2L as a promising target and stimulate research on UBAP2L-based future cancer therapies.
